# Practical considerations for data exploration in quantitative cell biology

**DOI:** 10.1242/jcs.263801

**Published:** 2025-04-07

**Authors:** Joanna W. Pylvänäinen, Hanna Grobe, Guillaume Jacquemet

**Affiliations:** ^1^ Turku Bioscience Centre, University of Turku and Åbo Akademi University, FI-20520 Turku, Finland; ^2^ Faculty of Science and Engineering, Cell Biology, Åbo Akademi University, FI-20520 Turku, Finland; ^3^ InFLAMES Research Flagship Center, University of Turku and Åbo Akademi University, FI-20520 Turku, Finland

**Keywords:** Data exploration, Data analysis, Microscopy, Data management

## Abstract

Data exploration is an essential step in quantitative cell biology, bridging raw data and scientific insights. Unlike polished, published figures, effective data exploration requires a flexible, hands-on approach that reveals trends, identifies outliers and refines hypotheses. This Opinion offers simple, practical advice for building a structured data exploration workflow, drawing on the authors' personal experience in analyzing bioimage datasets. In addition, the increasing availability of generative artificial intelligence and large language models makes coding and improving data workflows easier than ever before. By embracing these practices, researchers can streamline their workflows, produce more reliable conclusions and foster a collaborative, transparent approach to data analysis in cell biology.

## Teaser

**Summary:** We present practical guidelines for data exploration in quantitative cell biology that are designed to help yield reliable conclusions and promote a collaborative, transparent approach to data analysis.

## Introduction

The increasing complexity of imaging, genomics and proteomics data is transforming quantitative cell biology. The resulting surge in data scale and complexity demands sophisticated data science approaches to dissect cellular processes quantitatively (Kobayashi et al., 2022; Lange et al., 2024; Viana et al., 2023; Weisbart et al., 2024). By leveraging advanced computational tools, we can systematically manage, analyze and interpret vast datasets to reveal subtle, quantitative patterns and interactions that underpin cell dynamics.

Data exploration is a fundamental step in quantitative cell biology (Royle, 2020) that is not always considered or planned sufficiently. It bridges raw data and meaningful scientific insights, helping researchers make sense of their datasets and guiding them toward reliable conclusions. Unlike the polished visualizations of data in published papers, which are crafted for clarity and focus on delivering a single message, exploratory data analysis is about diving into the data from different angles, uncovering trends, spotting outliers and refining hypotheses. It should be a hands-on, flexible process. Although data exploration is essential in quantitative cell biology, practical guidance on structured data exploration is rare. For many researchers eager to apply data science methods to their biological data, building a robust exploratory data analysis workflow often means learning by trial and error. Moreover, as journals increasingly require source data files following the FAIR principles (Wilkinson et al., 2016), ensuring that data are well organized and annotated throughout the exploration phase can save considerable time in the long run. Finally, facilitating effortless data sharing within a research group depends on having clearly documented, accessible datasets at every stage.

Our lab uses live-cell and fixed imaging techniques to investigate how cancer cells interact with their microenvironment during metastasis (Follain et al., 2024 preprint; Jacquemet et al., 2017; Peuhu et al., 2022). At the same time, we continuously develop image analysis strategies to extract information from our images (Ershov et al., 2022; Hidalgo-Cenalmor et al., 2024; von Chamier et al., 2021). Our experience shows that robust data exploration workflows not only enhance our ability to extract meaningful quantitative insights but also facilitate the preparation of data for machine learning approaches. Here, we outline the guiding principles behind our ideal data exploration workflow. We provide eight practical recommendations that we apply in our lab to streamline data analysis. We also explain how generative artificial intelligence (AI) and large language models (LLMs) can enhance the accessibility of programming languages, making data exploration more efficient. Finally, we share our perspectives on the importance of optimizing data workflows.

Although these recommendations arise from our experience analyzing microscopy images, the underlying principles may broadly apply to various types of research. Many of these practices might seem obvious to data scientists, but they merit revisiting for anyone interested in refining their data handling practices. It is essential to emphasize that these recommendations are based on our personal experiences and opinions, with some aspects reflecting individual preferences rather than universal best practices.

## Core principles for our ideal data exploration workflow

Although creating a one-size-fits-all data exploration workflow is challenging, several core principles apply. Box 1 outlines key terminology related to data exploration, and Box 2 details common data types used in quantitative cell biology. Our ideal workflow includes the following guidelines.

**Box 1. Some useful terminology**

**Fig. 1.**
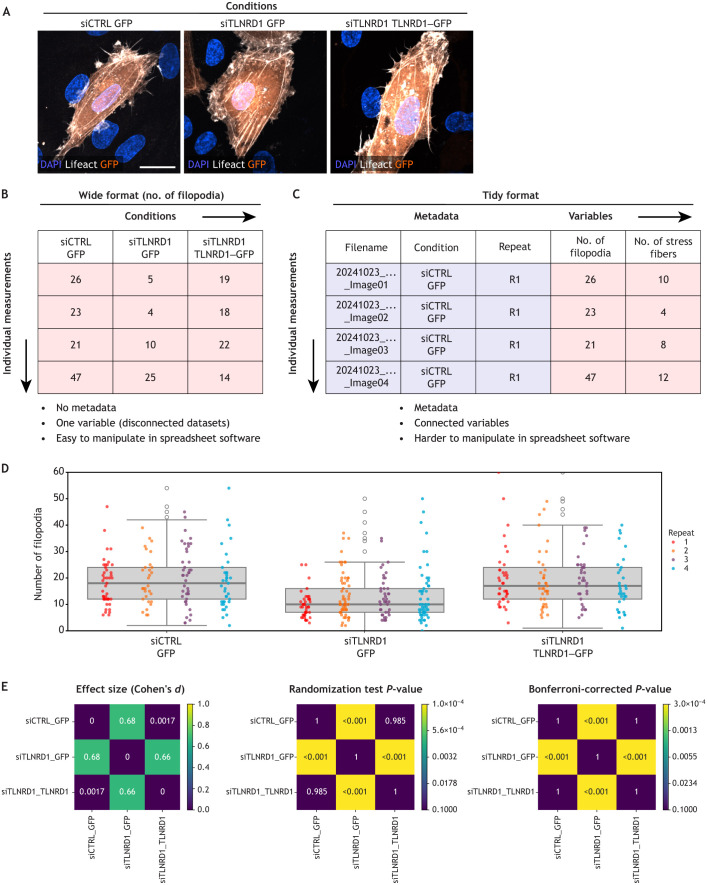
**From data organization to visualization – the role of tidy data in practical analysis.** (A–E) Here, we show data exploration and visualization for an experiment comparing the number of filopodia in control cells with that in cells treated with siRNA targeting TLNRD1 (siTLNRD1). (A) Human umbilical vein epithelial cells (HUVECs) expressing Lifeact–RFP under control (siCTRL, with GFP expression) or experimental (treated with siTLNRD1, with either GFP or TLNRD1–GFP expression) conditions. Cells were fixed, stained with DAPI and imaged using spinning disk confocal microscopy. Representative maximal intensity projections of fields of view are shown. Scale bar: 20 µm. Images were acquired as part of a previously published study (Ball et al., 2024). (B) Schematic of the wide data format, where data relating to the number of filopodia counted in each condition are stored across columns, making data more challenging to manipulate for downstream analyses. (C) Schematic of the tidy data format, where each variable (e.g. condition, filopodia count, repeat) is stored in a single column, and each observation occupies a row. This organization enables flexible data wrangling, statistical analysis and direct input into visualization tools such as box plots and heatmaps. (D) The number of filopodia per GFP-positive cell as shown in A was manually quantified across all conditions (four biological repeats, >60 fields of view per condition). Results are presented as box plots, where horizontal lines mark the median, boxes represent the interquartile range, and vertical whiskers extend to data points within 1.5× the interquartile range above and below the third and first quartiles, respectively. Dot plots showing each biological replicate uniquely color-coded are also presented as part of a SuperPlot (Lord et al., 2020) to visualize variability in the data. Importantly, the tidy data format was required to make this plot. (E) Pairwise condition comparisons using heatmaps. Three mirrored heatmaps, one displaying the effect size (Cohen's *d* values) and two displaying the statistical significance (*P*-values from randomization tests with and without Bonferroni correction). The plots in D and E were generated using Plot-Stats.

**Conditions:** in a typical experiment, different conditions are compared to assess the effects of a treatment, such as siRNA-mediated knockdown, on cellular behavior or phenotype (Fig. 1A). For example, comparing control cells to siRNA-treated cells expressing different constructs helps to identify treatment-specific responses.

**Variables:** we measure specific variables (or features) of the cells from these conditions to study these effects quantitatively. Here, a variable represents a measurable attribute or characteristic of the system; examples might include cell size, morphology (e.g. number of filopodia) or the fluorescence intensity of a particular marker.

**Biological and technical repeats:** experiments are conducted with multiple biological repeats to capture the biological variation. Biological repeats are defined as independent experimental replicates performed on different biological samples, and they are essential for verifying that observed effects are reproducible. Biological repeats should be distinguished from technical repeats, where measurements are taken from the same sample multiple times using identical conditions and equipment to assess the consistency and precision of the instrument used.

***n* numbers:** often used in figure legends, we use *n* numbers to document the number of measurements used to generate a graph. However, for transparency, it is important to clearly specify both the number of biological repeats and the number of data points per biological repeat to provide a complete picture of reproducibility. The definition of *n* can be somewhat controversial, and we encourage readers to refer to Lazic et al. (2018) for further context.

**Box 2. Data types in quantitative cell biology**

When working with quantitative data in cell biology, it is helpful to distinguish between data types, as they determine how information is organized, analyzed and visualized.

**Quantitative data** are data that can be measured numerically. This data type is divided into two categories: discrete data and continuous data.

**Discrete data** consist of countable, finite values, such as the number of cells in an image, the number of filopodia per cell or the sample size (*n* number). These values are whole numbers and cannot be subdivided.

**Continuous data** can take any value within a range and often obtained by making measurements. Examples include fluorescence intensity or cell size.

**Qualitative (categorical) data** are data that represent distinct groups or categories rather than numerical values. Experimental conditions such as target-specific siRNA treatment versus scrambled control siRNA treatment, or drug treatment versus vehicle control treatment are examples of categorical data.

In quantitative cell biology, we often analyze continuous measurements as a function of either continuous variables (e.g. time, drug concentration or spatial position) or qualitative conditions (e.g. control versus treated, wild type versus mutant). Understanding these distinctions helps in choosing the appropriate data processing and visualization techniques. In this article we primarily focus on handling and analyzing discrete and continuous data extracted from images, often measured as a function of qualitative conditions.

### Make it flexible

The workflow must allow easy processing of and access to data, as final results and interpretations often evolve throughout the exploration process. A data exploration workflow should adapt as new data are added, beginning with the first biological repeat and continuing incrementally until the dataset is complete.

### Incorporate visualization

Humans are visual creatures, and effective data exploration relies on generating clear, informative plots (Jambor, 2024). Visualizations enable quick interpretation of trends, identification of anomalies and observation of patterns that might be missed in tables of numbers. Visual representation of data is crucial for refining hypotheses and adapting experimental strategies as needed.

### Assess biological variability

We believe that consistently assessing biological variability and reproducibility is crucial to avoid premature conclusions. Unfortunately, standardized measures of repeatability and reproducibility are not readily available, making visual assessment a common but subjective approach (Weissgerber et al., 2015). In the case of discrete data, we find that SuperPlots, such as the one displayed in Fig. 1D, are especially useful for this purpose. They combine dot plots and box plots to display individual data points by biological repeat while also capturing overall trends (Goedhart, 2021; Lord et al., 2020). This approach provides a clear view of variability across repeats, which is essential for interpreting the robustness of experimental results.

### Keep the metadata

Tracking metadata during data analysis and exploration is crucial for understanding variability and ensuring reproducibility (Hammer et al., 2021; Sarkans et al., 2021). Some metadata, like timestamps and instrument settings, are typically automatically generated and saved during an experiment. In contrast, other key metadata, such as biological conditions, filenames and biological repeat numbers, might need to be manually recorded. Both types of metadata are essential for supporting transparent and reproducible data exploration and facilitating dataset sharing. Certain data handling practices, such as using the ‘tidy’ format (discussed below), can help streamline metadata organization and integration.

### Save and organize the results

Saving final results in an organized manner makes it easy to share data with group members and to edit visualizations further for presentations and publications. Store plots and other outputs systematically and consider saving visualizations as vector files (such as PDF or SVG files) so that they can be easily opened and edited with other software. Avoid deleting intermediate files, as doing so can lead to a significant waste of time and effort. These files often contain valuable insights and can serve as a foundation for future analyses.

## Guidelines to improve your data exploration capabilities

Considering the core principles described above, we have gathered eight pieces of advice that have improved our group's data exploration capabilities over the past few years.

### Learn R or Python to improve file and data handling

Spreadsheet software, like Microsoft Excel or LibreOffice, can be a helpful starting point, yet the limitations of such software might be constraining. Learning R or Python can be transformative if you want to enhance your data exploration workflow by eliminating repetitive manual tasks, such as automating the compilation of result files and creating plots. Although learning to write code may initially seem intimidating, we assure you that mastering an open-source coding language will be worthwhile. Moreover, with the support of LLMs, getting started is easier than ever – you don't need to be a coder to benefit from these tools. However, understanding the basics of R or Python and reading these languages will empower you to interpret, debug and customize LLM-generated code to suit your needs. In a later section of this article, we will provide more details on optimizing the use of LLMs in your workflow.

R and Python offer robust, open-source ecosystems for creating automated analysis pipelines that go far beyond the constraints of proprietary software. If your work involves image data, Python's extensive imaging and machine learning libraries make it an excellent choice (Virtanen et al., 2020; van der Walt et al., 2014; https://napari.org/). For single-cell RNA sequencing data, specialized packages available for R streamline genomic analyses (Hao et al., 2024). RStudio provides an integrated development environment for R, making code writing and debugging more intuitive, and easily connects with GitHub. Jupyter Notebooks – an interactive computing platform – support both Python and R, offering versatile spaces for exploration, visualization and sharing your analysis (Kluyver et al., 2016). Tools like Quarto (https://quarto.org/) further enhance this workflow by enabling seamless publishing and communication of results. Quarto provides a powerful, flexible, open-source framework for creating dynamic reports, presentations and websites directly from R, Python or Jupyter Notebooks.

Readers interested in learning R may find a recent article by Lawlor et al. useful (Lawlor et al., 2022). Those wanting to learn Python for image analysis should explore the Bio-image Analysis Notebooks (https://haesleinhuepf.github.io/BioImageAnalysisNotebooks/intro.html) and the Introduction to Bioimage Analysis (https://bioimagebook.github.io/) Jupyter books.

A practical way to get started is to use a small dataset, open a Jupyter Notebook (such as Google Colab; https://colab.google/) and interact with an LLM for guidance. Ask it how to conduct the analysis you need and have it explain the code as it is generated. This hands-on method will help you build confidence, develop problem-solving skills and refine your workflow.

### Adopt the ‘tidy’ format

How you structure and store your numerical data significantly impacts your ability to analyze, interpret and share it effectively (Hertz and McNeill, 2024). Two widely used formats in data organization are the ‘wide’ format and the ‘tidy’ format (Wickham, 2014). The wide format, commonly used in spreadsheet software, places conditions (Fig. 1A) across columns and organizes each variable in separate tables (Fig. 1B). The wide format is fully compatible with most spreadsheet software.

In contrast, the tidy format assigns each variable to a column and each observation to a row, with all data organized within a single table (Fig. 1C). Crucially, the tidy format allows essential metadata – such as filenames, conditions and biological repeats – to be stored as columns alongside other variables, making it easy to keep all critical information together in one place. Because of this, the tidy format enables the creation of SuperPlots that help assess biological variability and reproducibility (Fig. 1D).

The tidy format is also highly adaptable, as new measurements can be added as additional rows without disrupting the overall structure. This flexibility enables rapid and straightforward comparisons across conditions, accelerating data exploration and reducing the risk of manual errors. Additionally, by storing multiple variables per measurement as separate columns, the tidy format makes it easy to explore relationships between variables or to perform advanced analyses such as dimensionality reduction methods, including principal component analysis.

Although spreadsheet software may not fully support tidy data for figure creation or analysis, many popular tools – such as PlotsOfData and libraries in R and Python – work seamlessly with this data format (Hunter, 2007; Postma and Goedhart, 2019; Wickham, 2014). Despite these minor limitations, adopting the tidy format will greatly streamline your data exploration and provide a robust data-sharing framework. For additional tips on handling tabular data, we recommend a recent article by Hertz and McNeill (Hertz and McNeill, 2024).

### Stay consistent when acquiring and analyzing your data

In the context of bioimage data, this article does not provide guidance on key steps of the bioimaging pipeline, as excellent resources already exist. For such guidance, we encourage readers to draw from the extensive literature on experimental design (Senft et al., 2023; Wait et al., 2020), image acquisition (Reiche et al., 2022), image analysis (Lee et al., 2024), statistical methods (Lord et al., 2020; Makin and Orban de Xivry, 2019; Zweifach, 2024), experimental reporting (Aaron and Chew, 2021; Laine et al., 2021) and figure preparation (Jambor, 2023, 2024; Jambor et al., 2021). These references offer foundational guidance for strengthening any data exploration workflow. Within this framework, we believe two core principles can significantly improve the quality and reliability of analyses: designing appropriate controls and maintaining consistency between biological repeats.

First, designing and incorporating appropriate controls is essential for validating experiments. Positive and negative controls help confirm that experimental procedures function correctly and ensure that observed differences are meaningful. Building controls into your experiments better distinguishes between actual biological effects and technical artifacts (Senft et al., 2023).

Second, strive to maintain consistency between biological repeats. Automated analysis workflows depend on uniformity. Even minor discrepancies in how samples are prepared or imaged can introduce variability and complicate downstream analysis. For example, differences in exposure time, magnification or channel acquisition order can distort results. Standardizing these parameters across experiments ensures compatibility, enabling accurate, reproducible comparisons and leading to more robust conclusions.

### Use informative and standardized filenames

Adopting a clear naming convention from the start of a study keeps data organized and traceable throughout the analysis pipeline. For example, when working with image files, avoid vague labels like ‘image1’ or ‘sample2’; instead, opt for structured filenames that include details such as the date, condition, biological repeat number, staining and image number (for example, ‘20241023_Control_R1_Channel1_Channel2_Image01’). This approach makes each file uniquely identifiable. This metadata can be efficiently integrated into the dataset with the tidy data format (Fig. 1C). Be consistent with naming between repeats, as sometimes changes (such as using different names, using lower versus upper case, or interchangeably using dashes and underscores) can significantly complicate the automated analysis process and waste time. When collaborating on a project, we recommend discussing and agreeing on a standardized naming convention early on to ensure a smooth and efficient data analysis workflow.

Additionally, avoid using overly long filenames, spaces or special characters, as these can cause issues with certain software tools. For example, some Fiji (Schindelin et al., 2012) macros truncate long filenames, potentially omitting crucial information.

Design your analysis and exploration pipeline to incorporate filenames directly whenever possible. This approach simplifies data management and adds transparency to your workflow, ensuring that links between the measurements and the raw data remain intact. Implementing these practices can minimize variability, streamline data management and establish a solid foundation for reproducible and efficient data exploration.

### Think beyond the *P*-value

In the life sciences, *P*-values are often overemphasized as the ultimate indicator of (biological) significance. Reaching the threshold of *P*<0.05 can lead to a binary interpretation of results: either ‘significant’ or ‘not significant’. This focus on significance has fueled practices like *P*-hacking (such as testing until significance is achieved) (Head et al., 2015; Makin and Orban de Xivry, 2019) and an overemphasis on selecting the ‘correct’ statistical test. However, relying solely on *P*-values to assess biological relevance obscures the bigger picture in data exploration (Halsey et al., 2015).

Indeed, statistical significance does not necessarily equate to biological relevance. A statistically significant result does not reflect the biological importance of an effect, nor does a lack of statistical significance rule out meaningful differences. Statistical tests are useful but limited tools – they simply provide the likelihood that two data distributions are the same based on the available data (Box 3) (Halsey et al., 2015).

**Box 3. Some useful statistical terms**

**The null hypothesis:** the null hypothesis is the assumption that there is no real difference between the groups being compared.

***P*-value:** the *P*-value indicates the probability of obtaining a result as extreme or more extreme than that observed, assuming the null hypothesis is true. A *P*-value close to 0 suggests that the observed difference is unlikely due to chance. Conversely, a *P*-value close to 1 suggests that any observed difference is likely due to random variation. *P*-values do not measure the size or biological relevance of an effect.

**Cohen's *d* value:** Cohen's *d* is calculated by subtracting the mean of one group from the mean of another and dividing the result by the pooled standard deviation (Cohen, 2013). This standardization allows for comparisons across different studies or experiments, regardless of sample size. Higher absolute values of Cohen's *d* suggest larger differences, and thresholds for small (0.2), medium (0.5) and large (0.8) differences are commonly used for interpretation. However, we do not recommend using these thresholds rigidly.

**Randomization test:** a randomization test (also known as a permutation test) involves calculating the observed statistic (e.g. Cohen's *d*) between two groups. This is followed by random shuffling of the group labels many times (e.g. 10,000 permutations) to create a distribution of the statistic under the null hypothesis. The *P*-value is determined by the proportion of randomized statistics that are as extreme as or more extreme than the observed one. This method provides a significance level without relying on parametric assumptions, making it robust for various data types.

**Bonferroni-corrected *P*-value:** *P*-values can be adjusted to account for multiple comparisons using the Bonferroni correction, resulting in a Bonferroni-corrected *P*-value. This is calculated by multiplying the original *P*-value by the number of tests performed. Although this method reduces false positives, it is conservative and can increase the risk of false negatives.

In addition, *P*-values alone do not offer insight into the magnitude of the difference. To address this, we recommend computing relative or absolute effect sizes, such as Cohen's *d* value (Box 3; Fig. 1E), which quantitatively measures the difference between groups independently of the number of data points. When used together, *P*-values and effect sizes can indicate whether distributions differ and by how much, offering a more nuanced view that is invaluable during exploratory analysis (Goedhart, 2019 preprint). We have found that visualizing *P*-values and Cohen's *d* values as heatmaps creates an effective data exploration workflow for pairwise comparisons (Fig. 1E). This approach makes it easy to identify trends at a glance and provides a visually compelling overview of significance and effect size.

The choice of which statistical test to use when exploring your data depends on your dataset. During data exploration, we often start with a non-parametric approach, such as a randomization test, to avoid making assumptions about data distribution (Box 3). However, it is essential to explore other alternatives based on your data and to avoid common mistakes, such as using a small sample size, failing to correct for multiple comparisons or conflating correlation with causation (Makin and Orban de Xivry, 2019).

Another consideration is the tendency of *P*-values to decrease with larger sample sizes, often yielding very small *P*-values for large datasets (Gómez-de-Mariscal et al., 2021). One way to counter this is by calculating *P*-values based on the averages of each biological repeat, thereby decreasing the number of values to compare while ensuring equal weighting across repeats (Lord et al., 2020). This approach is efficient when you have a sufficient number of biological repeats. We find that non-parametric randomization tests can be robust enough to provide meaningful information without oversimplifying the data. In this case, ensuring that each biological repeat contributes equally to the analysis is essential. When one repeat contains significantly more data points, this imbalance can skew results and visualizations (Gómez-de-Mariscal et al., 2024). Resampling data to balance the number of points per repeat can be beneficial in such cases.

Ultimately, it is crucial to remember that statistical significance does not equate to biological relevance. Carefully use *P*-values and statistical tests as tools in your data exploration, and always consider the broader biological context of your findings.

### Normalize your data responsively

Data normalization can be a valuable tool in data exploration, especially since raw values can show variability between biological repeats. However, it is essential to approach normalization thoughtfully to preserve the underlying structure and variability of your data.

To start, always examine the raw values before applying any normalization. This initial assessment can reveal trends and inconsistencies that might be obscured by normalization and allows you to better understand your dataset without potential biases. When normalizing, we recommend using the average of the control condition within each biological repeat as a baseline. This method retains the relative variability within each repeat, helping you compare conditions while accounting for differences across repeats.

In cases where you only have one value per condition per repeat, avoid normalizing in a way that fixes the control to a value of 1 in each repeat, as this can mask control variability and distort interpretation. Instead, consider normalizing to the total sum of observations in each repeat. This approach is particularly helpful for analyzing western blots, as it preserves integrity of the biological variability of the control (Degasperi et al., 2014).

Be cautious when interpreting fold changes over a control, as increases often seem more exaggerated than decreases, leading to a skewed interpretation. We find that applying a log_2_ transformation to your data can mitigate this effect, making increases and decreases more comparable and interpretable. By applying normalization methods that account for these factors, you can make your data more comparable across conditions and repeats without losing sight of the biological variability.

### Take advantage of LLMs

LLMs can be powerful tools in building your data exploration workflow. They can assist in writing code for data wrangling, visualization and analysis, even for those who are not experienced in coding. LLMs have already been used effectively in image analysis (Lei et al., 2024; Royer, 2024). However, when using LLMs to generate code, it is crucial to learn how to read and understand the code to ensure accuracy and prevent errors in your data analysis. Indeed, although LLMs can generate code quickly, it is essential to understand and verify what they produce. Reviewing the code line by line, running tests and performing validations ensures that the output is accurate and error-free. This scrutiny will safeguard the integrity of your workflow.

LLMs can also provide valuable suggestions on the types of analyses you could perform on your data to answer specific questions. This capability is especially valuable as it allows you to transcend traditional disciplinary boundaries and explore methods from other fields. However, it is important to cross-validate these suggestions, as LLMs can hallucinate or provide incorrect information (Haase et al., 2024 preprint). Likewise, be aware that LLMs might re-implement functionality already available in established toolboxes and, therefore, may not always suggest the most straightforward option. Investigate existing well-tested resources before adopting newly generated solutions. Finally, be careful not to feed sensitive data to an LLM, as privacy may not be guaranteed.

**Fig. 2.**
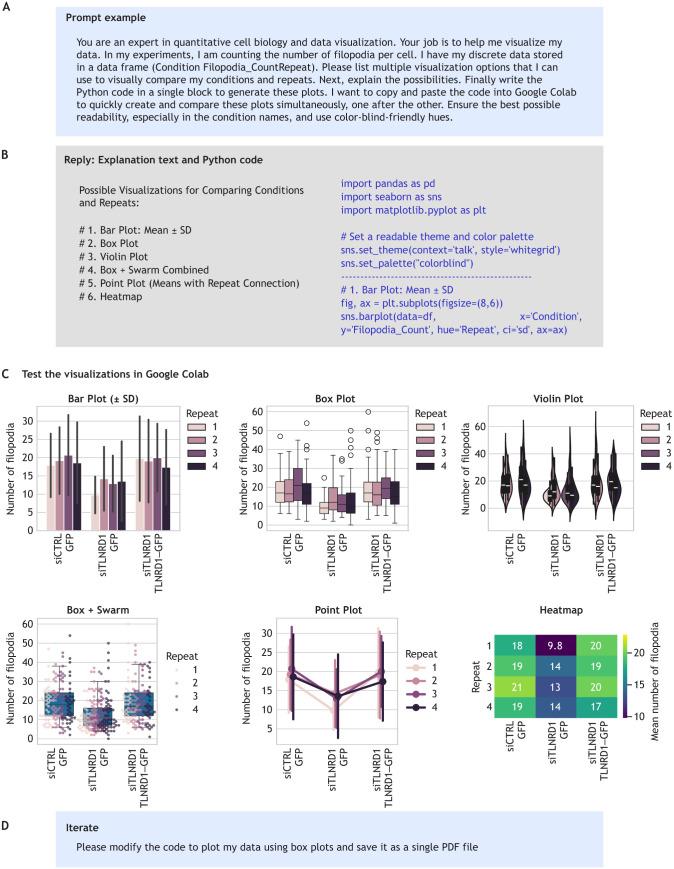
**Using LLMs to explore data visualization options.** (A) An example prompt provided to an LLM requesting visualization suggestions for a given dataset. (B) A summarized response from the LLM, illustrating how it proposes multiple visualization techniques (such as bar plots, box plots and violin plots) alongside explanatory text and example Python code. The code generated by the LLM can be copied directly into Google Colab, enabling rapid generation of the suggested visualization options. This approach allows you to identify the most appropriate and informative visualization method for your data. (C) Selected example plots are displayed (as generated). (D) An example of iterative refinement through additional prompts, demonstrating how the LLM output can be further tuned – such as by adjusting error handling, saving figures or modifying color schemes – to meet specific visualization needs. The LLM used here to make this figure was ChatGPT GPT-4o (OpenAI).

Another valuable application of LLMs is obtaining advice on various types of data visualization for your dataset (Fig. 2). They can provide meaningful suggestions and even generate code to help you compare different visualization approaches side by side, enhancing your exploratory analysis. You can then quickly assess which visualizations work best for your data and specific needs. By integrating LLMs into building your workflow, you can quickly identify various ways to represent and analyze your data during the exploration stage, making the process more efficient and comprehensive.

### Build your own data exploration workflow

Investing time in building a data exploration workflow tailored to your data types and personal preferences using the many tools available can greatly enhance efficiency and reproducibility. For example, our lab uses spreadsheet software (e.g. LibreOffice) and Jupyter Notebooks for data exploration. Jupyter Notebooks provide an interactive computing environment where users can write and execute code in sections (cells), combining text, visualizations and analysis in a single document (Kluyver et al., 2016). This format makes it easy to build and document workflows, run analyses step by step and share documented analysis pipelines. Jupyter Notebooks support multiple programming languages, including Python and R, making them versatile.

We are particularly fond of using Google Colab, a cloud-based implementation of Jupyter Notebooks. Google Colab allows users to run notebooks without requiring local setup or installation, making it particularly useful for those new to coding or working on shared projects. It provides free and paid access to cloud computing resources, which can be valuable for machine learning and large-scale data analysis (Mirdita et al., 2022; von Chamier et al., 2021). Additionally, Google Colab enables the creation of simple interactive forms, which improve user input and workflow customization (for an example, see https://colab.research.google.com/notebooks/forms.ipynb).

**Fig. 3.**
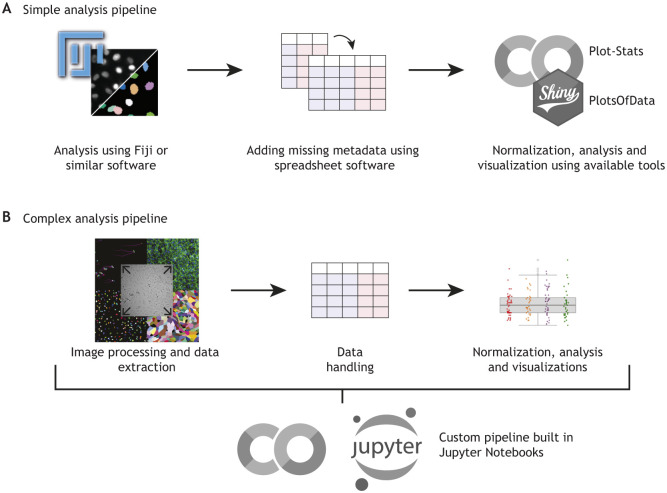
**Example of our current data analysis workflow.** (A) For simpler analysis pipelines, such as data generated using Fiji results tables, we store datasets in tidy format using spreadsheet software. We ensure that filenames, biological repeats and conditions are clearly labeled. To visualize the data, we either copy and paste it directly into Plot-Stats (which uses Google Colab) or use PlotsOfData Shiny apps (Goedhart, 2019 preprint; Postma and Goedhart, 2019) for immediate plotting and interpretation. (B) For more complex pipelines, such as those involving image data processed directly in Python, or when handling large, unwieldy datasets, we rely on a tailored Python script to load and process the data. These scripts may be developed with assistance from LLMs, making the process faster and more efficient. We then use Plot-Stats or integrate Plot-Stats code or other appropriate visualization methods directly into these specialized pipelines, ensuring a seamless and reproducible workflow.

The flexibility of Jupyter Notebooks, combined with the accessibility of Google Colab, allows us to design dynamic analysis workflows and easily share them when publishing papers (Follain et al., 2024 preprint; Gómez-de-Mariscal et al., 2024). Details on how we use these tools to build a workflow are outlined in Fig. 3.

To further streamline our data exploration, we have developed Plot-Stats (https://github.com/CellMigrationLab/Plot-Stats). This dedicated Google Colab Notebook collection converts data in wide format to tidy format, creates SuperPlots and computes key statistical measures (Fig. 3A), such as *P*-values and Cohen's *d*, displaying results as heatmaps such as those shown in Fig. 1E. Plot-Stats can also perform more complex analyses such as dimensionality reduction and clustering. Once a dataset is loaded, these notebooks can be readily adapted or expanded to accommodate new analyses. Although this approach suits the workflow in our lab, it might not fit every application perfectly. Establishing a similar, customizable strategy (Fig. 3B) can accelerate your data analysis and make it more reproducible and enjoyable.

## Why streamlining data exploration matters

Here, we share our perspectives on improving our data exploration practices to illustrate the value of these approaches and how this has shaped the advice we present in this article.

### Guillaume – group leader, Cell Migration lab

As an experimenter during my PhD and postdoctoral years, I took the time to dive deeply into my datasets, manually searching for trends and patterns. I relied heavily on spreadsheet software, spending days copying and pasting analysis results, generating plots and calculating *P*-values with *t*-tests. In hindsight, this approach was both limited and time consuming. A streamlined workflow would have allowed me to explore data more efficiently and rigorously. My perspective began to change when I discovered resources such as PlotsofData (Postma and Goedhart, 2019), blog posts by Joachim Goedhart on the Node (https://thenode.biologists.com/author/joachimg/) and ‘The Digital Cell’ book by Stephen Royle (Royle, 2020). Now, as a group leader, my time is divided across many tasks, and I can no longer delve into every dataset generated in the lab. Therefore, it is very helpful when lab members present and organize their data in a structured, digestible format that enables collaborative exploration. For example, we strive to store datasets on shared Google Drives, perform analyses in Google Colab notebooks and track changes in GitHub to maintain version control. This way, everyone involved in a study can access and contribute to the data analysis process. When we preprint a study, we release the code on GitHub and archive the code and key datasets on Zenodo to ensure accessibility and long-term preservation. This method supports efficient hypothesis generation, figure creation and data sharing in open repositories. In most projects, my role centers on data analysis rather than data acquisition, making a structured approach vital for effective collaboration. Streamlining data exploration is not only a time saver – it is essential for maintaining data integrity, consistency and, ultimately, the quality and reproducibility of our research.

### Joanna – postdoc, Cell Migration lab

Before joining the Cell Migration lab, I worked as a project researcher in multiple research groups. Usually, the datasets I analyzed were small, often only comparing two conditions. The go-to data analysis tool used by these research groups was usually Excel. Similarly to Guillaume, I had to go through multiple copy–paste steps to be able to plot the data. I was always uncomfortable with this, as I noticed that copying and pasting data often resulted in user errors that affected the analysis. To avoid this, I started creating pre-programmed Excel templates with written-in functions that automatically normalized and plotted my data. That was sufficient for me then, although I was limited to simple plots such as bar charts. After joining the Cell Migration lab, I started working with larger datasets, such as those from cell migration experiments generated by my group members. With these much more complex datasets, my analysis skills felt limited. I started to work with new tools such as FiloMap (Jacquemet, 2023) and CellTracksColab (Gómez-de-Mariscal et al., 2024), which introduced me to R and Python programming, respectively. As the need to analyze our data expanded, so did our need to share the analysis pipelines easily with other group members. Therefore, I started writing Python-based analysis notebooks using LLMs to directly process images, compile the results and analyze the data to match the needs of each project. Using this strategy, I can interactively share these notebooks with other group members to facilitate data analysis, which has significantly improved the ease of internal collaboration and reproducibility of our analysis, streamlining the overall process. I found this revolutionary: I was no longer limited to analysis using different kinds of software in sequence.

### Hanna – postdoc, Cell Migration lab

I was lucky enough to be introduced to R as a master's student and quickly learned the advantages of rapidly and reproducibly generating graphs from my measurements. Because tools like Fiji (Schindelin et al., 2012) produce consistent output formats across experiments, creating reusable code for data analysis and visualization became straightforward. By streamlining my workflow early on, I now only need to make minimal adjustments to the code for each new experiment, saving considerable time and effort. My current goal is to make the resulting data and analysis pipeline more easily shareable among researchers. To achieve this, I'm establishing standardized ‘landmarks’ at key points in the pipeline. For example, I maintain raw data in TIFF format, apply a clear and consistent naming scheme to files and attach well-documented metadata. Measurements are stored in a CSV file following the tidy data principles, ensuring that variables and observations are easily distinguishable. Likewise, plots are saved as vector graphics (such as PDF files) with standardized labels and annotations. This approach means that intermediate steps – such as choosing a particular microscope or data wrangling tool – can remain flexible and cater to individual preferences, but the data remain shareable and understandable at every stage. By setting these standards early, it becomes easier for current and future collaborators to understand the provenance of the data, apply their analyses and ultimately build upon the work.

## Future perspective

As data in quantitative cell biology continue to grow in complexity and volume, the importance of effective data exploration will only increase. The advice presented in this article enables researchers to move beyond the limitations of using spreadsheet-only software and progressively adopt more advanced tools for data exploration. Mastering these steps will enable researchers to naturally incorporate additional processes, such as image analysis, directly into workflows, further enhancing efficiency.

For students and seasoned researchers alike, developing robust data exploration workflows will serve you well throughout your career. For students, these skills lay a strong foundation for future research endeavors, equipping them with tools that will remain valuable as technology evolves. For group leaders, incorporating these tools into the lab will streamline team efforts and enable better management and oversight of the data generated. The next step is integrating data exploration practices seamlessly with data management and archiving platforms. In this space, tools such as OMERO and the Image Data Resource offer interesting opportunities (Allan et al., 2012; Williams et al., 2017).

Looking ahead, we must remain adaptable, continuously update our skill sets and invest time in learning new tools, adopting best practices and fostering a culture of collaboration. By embracing these strategies, we can collectively enhance the quality and impact of our research, driving the field forward.
